# Transcriptomic Profiling Reveals Discrete Poststroke Dementia Neuronal and Gliovascular Signatures

**DOI:** 10.1007/s12975-022-01038-z

**Published:** 2022-05-31

**Authors:** Rachel Waller, Yoshiki Hase, Julie E. Simpson, Paul R. Heath, Matthew Wyles, Rajesh N. Kalaria, Stephen B. Wharton

**Affiliations:** 1 Sheffield Institute for Translational Neuroscience, University of Sheffield, 385A Glossop Road, Sheffield, S10 2HQ UK; 2 Translational and Clinical Research Institute, Newcastle University, Newcastle upon Tyne, NE4 5PL UK

**Keywords:** Neurons, Gliovascular unit, Poststroke dementia, Transcriptome, Vascular dementia

## Abstract

Poststroke dementia (PSD) is associated with pathology in frontal brain regions, in particular dorsolateral prefrontal cortex (DLPFC) neurons and white matter, remote from the infarct. We hypothesised that PSD results from progressive DLPFC neuronal damage, associated with frontal white matter gliovascular unit (GVU) alterations. We investigated the transcriptomic profile of the neurons and white matter GVU cells previously implicated in pathology. Laser-capture microdissected neurons, astrocytes and endothelial cells were obtained from the Cognitive Function After Stroke cohort of control, PSD and poststroke non-dementia (PSND) human subjects. Gene expression was assessed using microarrays and pathway analysis to compare changes in PSD with controls and PSND. Neuronal findings were validated using NanoString technology and compared with those in the bilateral common carotid artery stenosis (BCAS) mouse model. Comparing changes in PSD compared to controls with changes in PSND compared to controls identified transcriptomic changes associated specifically with dementia. DLPFC neurons showed defects in energy production (tricarboxylic acid (TCA) cycle, adenosine triphosphate (ATP) binding and mitochondria), signalling and communication (MAPK signalling, Toll-like receptor signalling, endocytosis). Similar changes were identified in neurons isolated from BCAS mice. Neuronal findings accompanied by altered astrocyte communication and endothelium immune changes in the frontal white matter, suggesting GVU dysfunction. We propose a pathogenic model in PSD whereby neuronal changes are associated with frontal white matter GVU dysfunction leading to astrocyte failure in supporting neuronal circuits resulting in delayed cognitive decline associated with PSD. Therefore, targeting these processes could potentially ameliorate the dementia seen in PSD.

**Supplementary Information:**

The online version contains supplementary material available at 10.1007/s12975-022-01038-z.

## Introduction

Having a stroke over the age of 65 years old doubles the chance of developing dementia [1], while around 25% of stroke survivors develop progressive cognitive decline more than 3 months poststroke with the majority satisfying criteria for vascular dementia [2]. Previous pathological investigations in the Cognitive Function After Stroke (CogFAST) cohort, a longitudinal study of dementia incidence following stroke in the over 75’s [3], have shown that progressive cognitive decline, leading to poststroke dementia (PSD), is associated with pathology remote from the infarct, particularly in the frontal white matter. The infarcts themselves may be small so that direct infarct damage per se does not explain the progressive dementia. The association of PSD with pathology in frontal brain regions, specifically the dorsolateral prefrontal cortex (DLPFC) implicates damage to anterior cognitive circuits (ACC) involved in impaired executive function, which is a common characteristic of vascular dementia [4, 5].

Pathological changes have been identified in both the glutamatergic pyramidal neurons of the DLPFC and the astrocytes that form the gliovascular unit (GVU) of the frontal white matter in those stroke survivors who proceed to PSD compared to those that do not develop dementia [6, 7]. DLPFC neurons project to the dorsolateral head of the caudate, important for executive function incorporating anticipation, planning, goal setting and flexible thinking. Pyramidal neuronal cell volumes are reduced by 30–40% in layers III and V of the DLPFC of PSD subjects compared to poststroke non-dementia (PSND) and control (no dementia or stroke) subjects. This reduction in cellular volume directly correlates with an increase in neurofilament protein SMI-31 immunoreactivity in layer III of the DLPFC of PSD subjects. These neuronal changes are not associated with neurofibrillary tangle pathology suggesting a vascular origin of the observed neuronal atrophy [7] that differs between cortical layers. Additionally, an increased number of clasmatodendritic astrocytes, immunopositive for fibrinogen, have been identified within the frontal white matter of PSD subjects, indicative of blood brain barrier (BBB) disruption [6]. Astrocytic end-feet also show stripping of aquaporin-4, further evidence of pathology of the frontal WM GVU in PSD [6]. Gliovascular dysfunction is a key contributor to neuronal injury following ischaemic stroke and in other neurodegenerative diseases, with neuronal cell death, glial cell activation, BBB disruption and resulting serum protein accumulation and inflammatory response being associated with disease progression [8, 9]. In addition to gliovascular changes in PSD, there is a higher burden of white matter hyperintensities and diffuse myelin changes in PSD compared to PSND subjects [6].

The current study aimed to identify the cellular and molecular basis of PSD by investigating the transcriptomic profile of the cells that have been implicated by previous pathological studies. Our study focused particularly on the pyramidal neurons of the DLPFC, but we also compared this with changes in capillary endothelial cells and astrocytes of the frontal white matter. We hypothesised that PSD results from progressive neuronal damage in the DLPFC that is remote from the stroke and that this may be associated with alterations in the GVU of frontal white matter with BBB disruption, astrocyte dysregulation and a neuroinflammatory response.

## Materials and Methods

### Human Central Nervous System (CNS) Tissue

Human post-mortem brain tissue was obtained from the Newcastle CogFAST Study. Ethical approval was obtained from the Joint Ethics Committee of Newcastle and North Tyneside Health Authority, Newcastle University and the University of Northumbria at Newcastle, and participants gave written informed consent in accordance with the declaration of Helsinki. Cognitive function and dementia tests including Mini-Mental State Exam (MMSE) (PSD average: 13; PSND average: 24), CAMCOG (PSD average: 50; PSND average: 88), Consortium to Establish a Registry for Alzheimer’s Disease (CERAD) (PSD average: 0.7; PSND average: 1.0) and Braak staging (PSD average: 1.9; PSND average: 2.1) were considered when selecting participants as part of the current study (Supplementary Table 1). Full CogFAST study cohort characteristics have been described previously [3, 10]. Generally, most stroke survivors in the CogFAST study considered clinically demented by the time of their death showed a high burden of vascular pathology in the general absence of Alzheimer’s type pathology [11]. It has been acknowledged that stroke survivors may not be completely free of Alzheimer’s pathology of amyloid-β burden but most stroke survivors over the age of 75 years present with significant vascular dementia over Alzheimer’s [12]. Snap frozen DLPFC and frontal white matter samples (*n* = 10 for each group) were collected from PSD, PSND and control subjects (no dementia or stroke). All PSD and PSND samples were from brains with infarcts of < 5 mm remote from the DLPFC and frontal white matter investigated in this current study. Where possible, cases were selected equally based on age and sex (Table 1, Supplementary Table 2). The quantity of RNA from each sample was analysed using a NanoDrop 1000 spectrophotometer (Thermoscientific, UK) and the quality of RNA was assessed on a 2100 Bioanalyzer (Agilent, Palo Alto, CA, USA). The RNA integrity number (RIN) of each frozen tissue block was assessed prior to laser capture microdissection (LCM) using a previously reported method [13, 14]. The mean pre-LCM RIN of the full set of DLPFC samples was 2.4, standard deviation (SD) 1.02; range 1.0–7.1 comprising of the PSD group (2.3, SD 0.24; range 1.9–2.7), PSND group (2.0, SD 0.53; range 1.0–2.6) and control group (3.0, SD 1.62; range 1.6–7.1). The mean brain pH of the samples was 6.44, SD 0.40; range 5.77–7.19 comprising of the PSD group (6.39, SD 0.43; range 5.77–7.03), PSND group (6.45, SD 0.46; range 6.00–7.04) and control group (6.49, SD 0.37; range 5.87–7.09). The mean post-mortem interval (PMI) time of the samples was 41 h SD 25.24 h; range 12–98 h comprising of the PSD group (45 h, SD 28.07 h; range 12–88 h), PSND group (47 h, SD 22.99 h; range 19–81 h) and control group (38 h, SD 26.33; range 15–98 h). The mean age at death of the samples was 85 years, SD 8.3 years; range 72–99 years comprising of the PSD group (89 years, SD 6.9 years; range 75–97 years), PSND group (85 years, SD 6.0 years; range 78–99 years) and control group (80 years, SD 9.4 years; range 72–96 years).

**Table 1:** CogFAST cohort: case number, age, gender, post-mortem delay, brain pH and cause of death

						**DLPFC sample**			
**Case**	**Age (yr)**	**Sex**	**PMI (h)**	**pH**	**Cause of death**	**Pre-LCM RIN**	**Neu RIN**	**Ast RIN**	**End RIN**
1-Con	72	M	17	6.14	Oesophageal adenocarcinoma	2.4	3.1	2.5	n/a
2-Con	78	F	23	6.54	Metastatic oesophageal carcinoma with broncho-oesophageal fistula	2.2	2.7	n/a	2.4
3-Con	72	F	27	5.87	Mesothelioma with metastases	2.5	3.0	n/a	2.0
4-Con	74	F	67	6.93	Heart failure and lung cancer	3.3	n/a	n/a	2.4
5-Con	74	F	53	6.43	Left ventricle failure. Ischaemic heart disease	2.4	3.1	2.2	n/a
6-Con	94	F	15	6.19	Metastatic cancer	1.6	2.6	2.3	n/a
7-Con	78	F	34	7.09	Bronchopneumonia	2.3	n/a	2.4	n/a
8-Con	89	F	98	6.72	Peritonitis	2.9	2.5	2.2	n/a
9-Con	73	M	25	6.45	Peritonitis	7.1	2.7	2.5	2.2
10-Con	96	F	29	6.58	Acute limb ischaemia	2.4	n/a	2.2	2.4
1-PSND	81	F	27	7.17	Acute left sided cerebral infarct	1.0	2.6	2.6	n/a
2-PSND	88	M	42	6.14	Bronchopneumonia/bronchitis	2.4	3.2	1.9	n/a
3-PSND	83	F	81	n/a	Aspiration pneumonia	1.6	2.6	2.5	3.5
4-PSND	84	M	50	6.29	Car accident—traumatic injuries	2.1	2.6	n/a	n/a
5-PSND	78	F	23	6.29	Acute cardiac arrhythmia	2.3	2.8	2.3	n/a
6-PSND	82	M	34	7.04	Heart arrhythmia	1.1	n/a	2.3	n/a
7-PSND	85	M	19	n/a	Heart failure	2.0	n/a	2.2	n/a
8-PSND	86	F	75	n/a	Not known	2.6	3.7	2.4	2.4
9-PSND	92	F	43	6.00	Cerebrovascular accident	1.9	2.8	n/a	n/a
10-PSND	99	M	76	6.22	Pneumonia	2.3	2.8	2.5	1.9
1-PSD	96	M	45	6.13	Acute/chronic gastro intestinal bleeding	2.7	2.9	2.3	2.4
2-PSD	88	F	71	5.77	Myocardial infarct	2.4	2.5	2.3	n/a
3-PSD	82	M	19	6.73	Aspiration pneumonia	2.6	2.5	2.3	n/a
4-PSD	93	M	30	6.03	Left sided cerebral infarct	2.3	2.2	2.3	n/a
5-PSD	87	F	23	6.95	Bronchopneumonia	2.4	2.6	2.1	n/a
6-PSD	75	M	24	5.97	Pneumonia	2.4	n/a	2.6	2.3
7-PSD	89	F	81	6.21	Asystole	2.2	n/a	n/a	n/a
8-PSD	96	M	64	6.49	Frailty of old age, dementia	2.2	2.6	2.3	n/a
9-PSD	97	F	88	7.03	Sepsis	1.9	2.8	2.3	1.1
10-PSD	91	M	12	6.55	Aspiration pneumonia	n/a	n/a	2.4	2.0

Key: *Ast*, astrocytes; *Con*, control; *DLPFC*, dorsal lateral prefrontal cortex; *End*, endothelial cells; *F*, female; *h*, hour; *M*, male; *n/a*, data not available; *Neu*, neurons; *PMI*, post-mortem interval; *PSD*, poststroke dementia; *PSND*, poststroke non-dementia; *RIN*, RNA integrity number; *yr*, year

### Laser Capture Microdissection and RNA Extraction of Neurons, Astrocytes and Endothelial Cells

Toluidine blue histologically stained pyramidal neurons [15, 16], glial fibrillary acidic protein-positive (GFAP^+^) astrocytes and collagen IV-positive (Coll-IV^+^) endothelial cells [14, 17] were isolated from each case using an Arcturus PixCell II LCM system (Thermoscientific, UK). Total RNA (approximately 50 ng/cell type) was extracted from each isolated enriched group of cells using the Arcturus PicoPure RNA isolation kit, according to the manufacturer’s instructions (Thermoscientific, UK) (Supplementary Fig. 1). The quantity and quality of the extracted RNA were determined using a NanoDrop 1000 spectrophotometer (Thermoscientific, UK) and a 2100 Bioanalyzer, respectively (Agilent, USA). Only those samples with a RIN of > 2.0 post-LCM or in the absence of a RIN, samples not showing a peak shift towards a shorter fragment size representing a more degraded sample on a Bioanalyzer trace, were taken forward for microarray analysis.

### RNA Amplification and Microarray Hybridisation

The target labelled mRNA was prepared following the GeneChip WT Pico amplification protocol (Applied Biosystems, ThermoFisher, UK). In brief, RNA amplification was achieved using low-cycle PCR followed by amplification using T7 in vitro transcription. The cRNA was converted to biotinylated sense-strand DNA hybridisation targets. Approximately 5.5 μg amplified cDNA was fragmented, labelled and hybridised to Clariom S Array chips for 16 h at 45 °C in a rotating oven at 60 rpm. Using the Fluidics Station 400 and GeneChip Operating System, the hybridisation cocktail was removed, and a series of stringency washing steps are followed to remove any unbound DNA, before each microarray was stained. Each microarray chip was scanned using the GeneChip 3000 scanner to determine the fluorescence intensity of hybridised transcripts.

### Microarray Analysis

Transcription Analysis Console (TAC) was used for quality control and analysis of the data, the individual sample. CEL files were imported into TAC version 4.0.1 (Applied Biosystems, USA) and several quality control (QC) parameters were checked for each sample including Poly-A RNA labelling controls, hybridisation controls, positive vs. negative area under the curve (AUC) and signal intensity to identify any outlier samples. Samples that passed QC were normalised to the median of all genes, prior to statistical analysis, to identify differentially expressed genes (DEGs). Transcripts were defined as being significantly differentially expressed if they showed a *p*-value of < 0.05 (unpaired *t*-test). DEGs were classified according to their biological function using the Database for Annotation Visualisation and Integrated Discovery (DAVID) [18, 19] using Kyoto Encyclopedia of Genes and Genomes (KEGG) pathway analysis to identify specific pathways while Functional Annotation Clustering (FAC) was used to cluster together functionally similar genes. In the case of a significant result, the *p* value was adjusted for multiple testing using the Bonferroni correction method and considered significant if *p* < 0.05.

### Neuronal Gene Expression Validation Using NanoString nCounter Analysis

Gene expression validation was performed on 14 samples consisting of 7 control and 7 PSD neuronal samples from the original cohort (Supplementary Table 3). Neuronal enriched RNA served as input into NanoString’s low RNA input amplification assay, designed to produce sufficient target for detection in an nCounter hybridisation assay. Multiplexed target enrichment (MTE) was achieved through the conversion of samples with low RNA concentration into cDNA, which was then amplified using target-specific primers. In brief, approximately 10 ng RNA was reverse transcribed using the following conditions: 10 min at 25 °C, 60 min at 42 °C and 5 min at 85 °C. For this work, NanoString human neuropathology gene expression panels were used for validation based on the initial microarray findings (NanoString Technologies, USA). Therefore, for MTE amplification 5 × dT Amp master mix with neuropathology panel gene specific primers were used. Following denaturation for 10 min at 95 °C, the products were amplified for 10 cycles under the following conditions, 15 s at 95 °C and 4 min at 60 °C.

Gene expression analysis was performed on the nCounter SPRINT Profiler system (NanoString Technologies, USA) according to manufacturer’s instructions and data analysed using the NanoString nSolver analysis v3.0 software (NanoString Technologies, USA). In brief hybridisation between target sample and reporter-capture probes pairs was performed for 22 h at 65 °C. Post hybridisation processing steps were carried out and excess probes removed. The probe/target complexes were aligned and immobilised on the nCounter cartridge. Image acquisition and data processing was carried out as per manufacturer’s protocol. Firstly, all data was background adjusted using internal negative controls followed by technical normalisation using internal positive controls. Data was then normalised to account for the variation in sample concentration and/or quality using the mean expression of the most stable housekeeping genes across samples and cartridges. Gene expression was calculated by counting the number of times each barcoded gene was detected above the defined background threshold and downstream differential gene expression between control and PSD cases was completed in the NanoString nSolver v3.0 software.

### Neuronal Gene Expression in the BCAS Mouse Model

For additional validation and comparison, the bilateral carotid artery stenosis (BCAS) mouse model [20] that replicates features of vascular dementia was used alongside control sham animals to characterise the neuronal transcriptomic changes in the frontal cortex (full animal procedures, Supplementary Methods 1). Toluidine blue stained cryosections from 5 BCAS and 5 Sham animals were subjected to LCM to isolate neurons from the frontal cortex of each animal as previously described [15]. Target labelled mRNA was prepared following the GeneChip WT Pico amplification protocol (Applied Biosystems, ThermoFisher, UK) and mouse Clariom S Arrays were used to investigate the neuronal gene changes across the two groups. Within TAC, transcripts were considered significantly, differentially expressed if they had a minimum fold change (FC) of plus or minus 1.2 and *p*-value of < 0.05 (unpaired *t*-test). The annotated gene lists were exported into DAVID where KEGG pathway analysis and FAC was carried out. Analysis was completed on each unique combined gene list (up- and downregulated genes) alongside analysis from the separate distinct up- and downregulated gene lists.

### Data Availability

The data that support the findings of this study are openly available in Gene Expression Omnibus at https://www.ncbi.nlm.nih.gov/geo/, reference number GSE186798.

## Results

### Gene Expression Changes in Neurons, Astrocytes and Endothelium

**Fig. 1:**
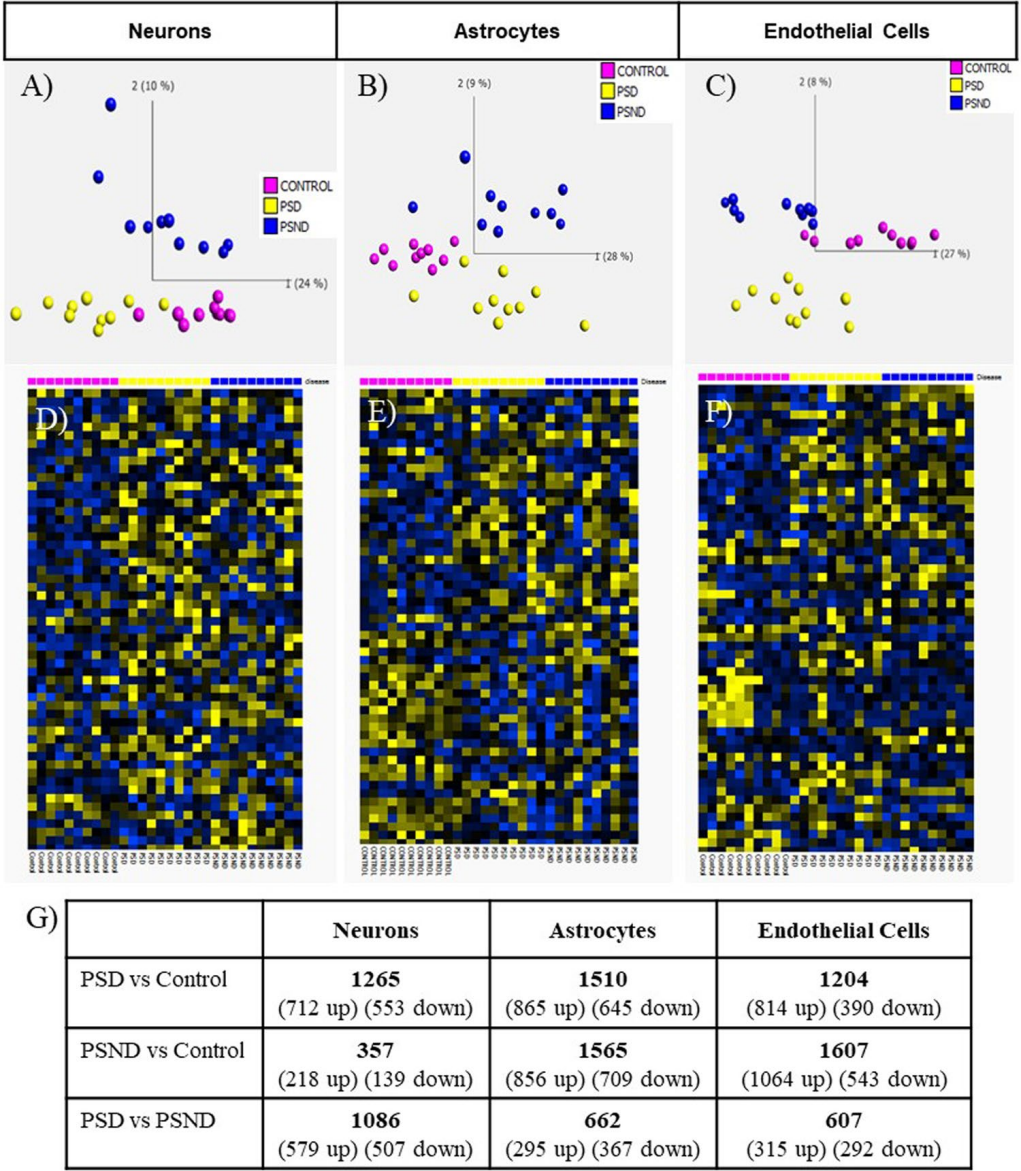
Gene expression analysis across cell types. Principal component analysis (PCA) reveals clear separation of control, poststroke dementia (PSD) and poststroke non-dementia (PSND) cases based on differential gene expression in neurons (A), astrocytes (B) and endothelial cells (C). Heatmap representation of differential gene expression in neurons (D), astrocytes (E) and endothelial cells (F). Transcription Analysis Console (TAC) analysis shows significantly expressed genes across patient comparison and cell types (G) (PSD; poststroke dementia, PSND; poststroke non-dementia)

Quality control measures were applied to each microarray-generated dataset in TAC. Based on gene expression alone, a clear separation of the different patient groups was confirmed for all three cell types using principal components analysis (PCA) (Fig. 1A–C) and heatmaps (Fig. 1D–F). All significantly DEGs (*p* < 0.05) between patient group comparisons, i.e. PSD vs control, PSND vs control and PSD vs PSND for each cell type were tabulated (Fig. 1G and Supplementary Table 4). In total from the neuronal data generated, 784 genes (PSD vs control), 193 genes (PSND vs control) and 608 genes (PSD vs PSND) were specific and exclusive to each patient group comparison. From the astrocyte data, 817, 767 and 363 genes respectively were specific and exclusive to each patient group comparison. While from the endothelial cell data, 681, 1048 and 352 genes were specific and exclusive to each patient group comparison.

These specific and exclusive gene lists from each patient comparison (PSD vs control, PSND vs control and PSD vs PSND) and cell type (neuron, astrocyte, and endothelial cells) were exported into DAVID where KEGG pathway analysis and FAC was carried out. Analysis was completed on each separate distinct up- and downregulated gene list alongside analysis from each unique combined gene list (up- and downregulated genes) (Supplementary Tables 5 and 6).

### The Neuronal Gene Expression Profile Is Significantly Altered in PSD Compared to PSND

**Fig. 2:**
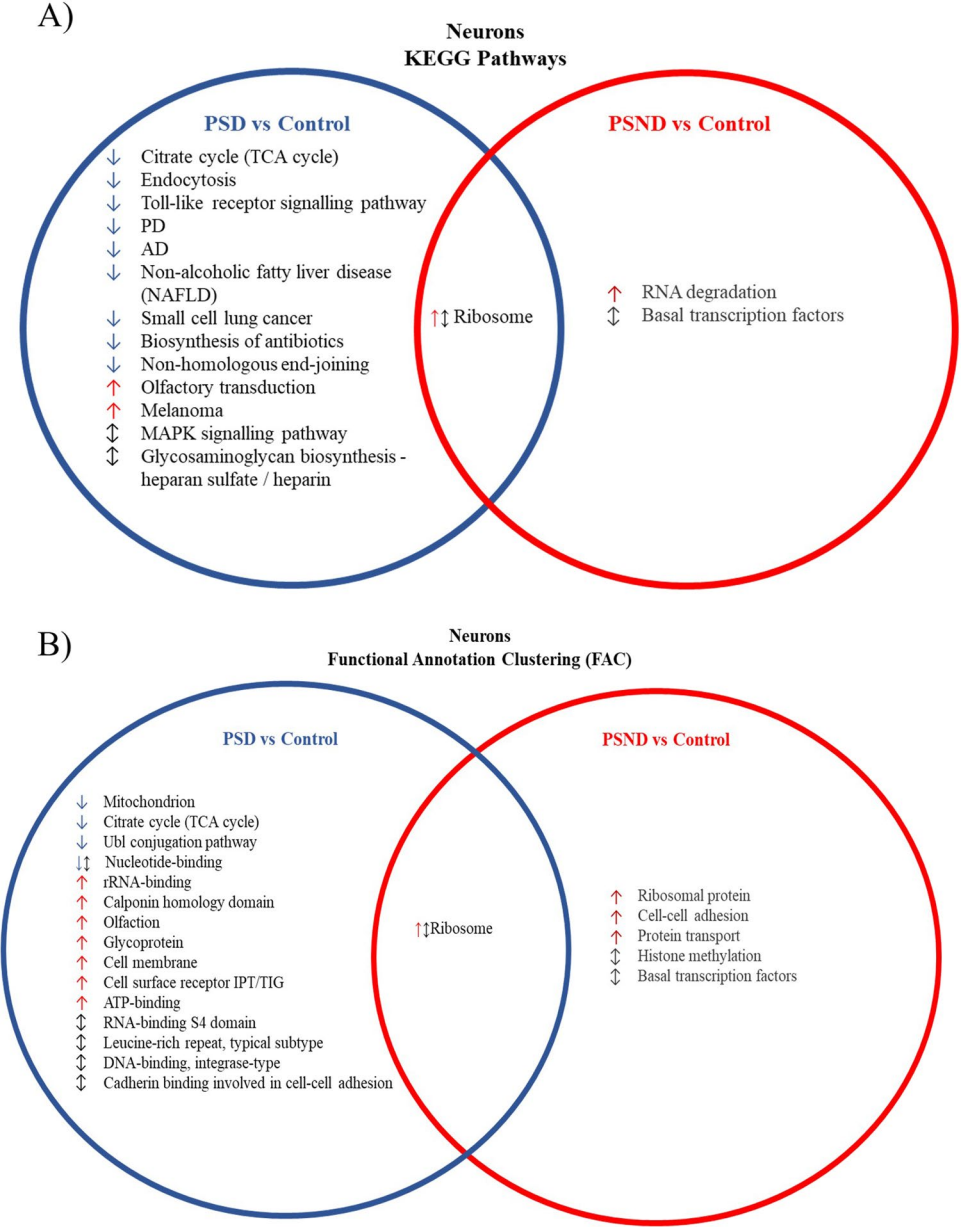
Comparing neuronal KEGG pathway analysis and functional annotation clustering associated with having a stroke (PSND vs. control) and having dementia resulting from a stroke (PSD vs. control) (↓ down regulated gene list, ↑ red arrow, upregulated gene list, ↕ up- and downregulated gene lists). (PSD, poststroke dementia; PSND, poststroke non-dementia)

Our experiments and analysis focused particularly on changes in pyramidal neurons of the DLPFC. To identify gene expression and pathway changes that might specifically be related to the development of dementia poststroke, we compared gene changes that were identified in PSD cases versus controls with those seen in PSND cases vs controls. Only 193 DEGs occurred in PSND neurons compared to control neurons. In contrast, 784 DEGs were identified in PSD compared to control neurons, including KEGG pathways associated with neuronal metabolism/mitochondrial changes (ribosome [25 DEGs, p 3.39E-06], Parkinson’s disease [9 DEGs, p 4.47E-02], Alzheimer’s disease [11 DEGs, p 1.88E-02], tricarboxylic acid (TCA) cycle [5 DEGs, p 9.13E-03]) and signalling changes (MAPK signalling [25 DEGs, p 2.87E-02], endocytosis [13 DEGs, p 3.68E-02] and Toll-like receptor signalling [8 DEGs, p 2.83E-02]) (Fig. 2A). Additionally, FAC carried out in DAVID identified the mitochondrion cluster (enrichment score (ES); 1.68, 44 genes, p 6.90E-03) and neuronal metabolism-related clusters including nucleotide binding (ES; 1.90, 69 genes, p 9.60E-04), glycoprotein (ES; 1.73, 181 genes, p 2.20E-03) and adenosine triphosphate (ATP) binding (ES; 1.31, 61 genes, p 1.6E-02) (Fig. 2B).

### Poststroke Dementia Neuronal Changes Verified by NanoString nCounter Analysis

**Fig. 3:**
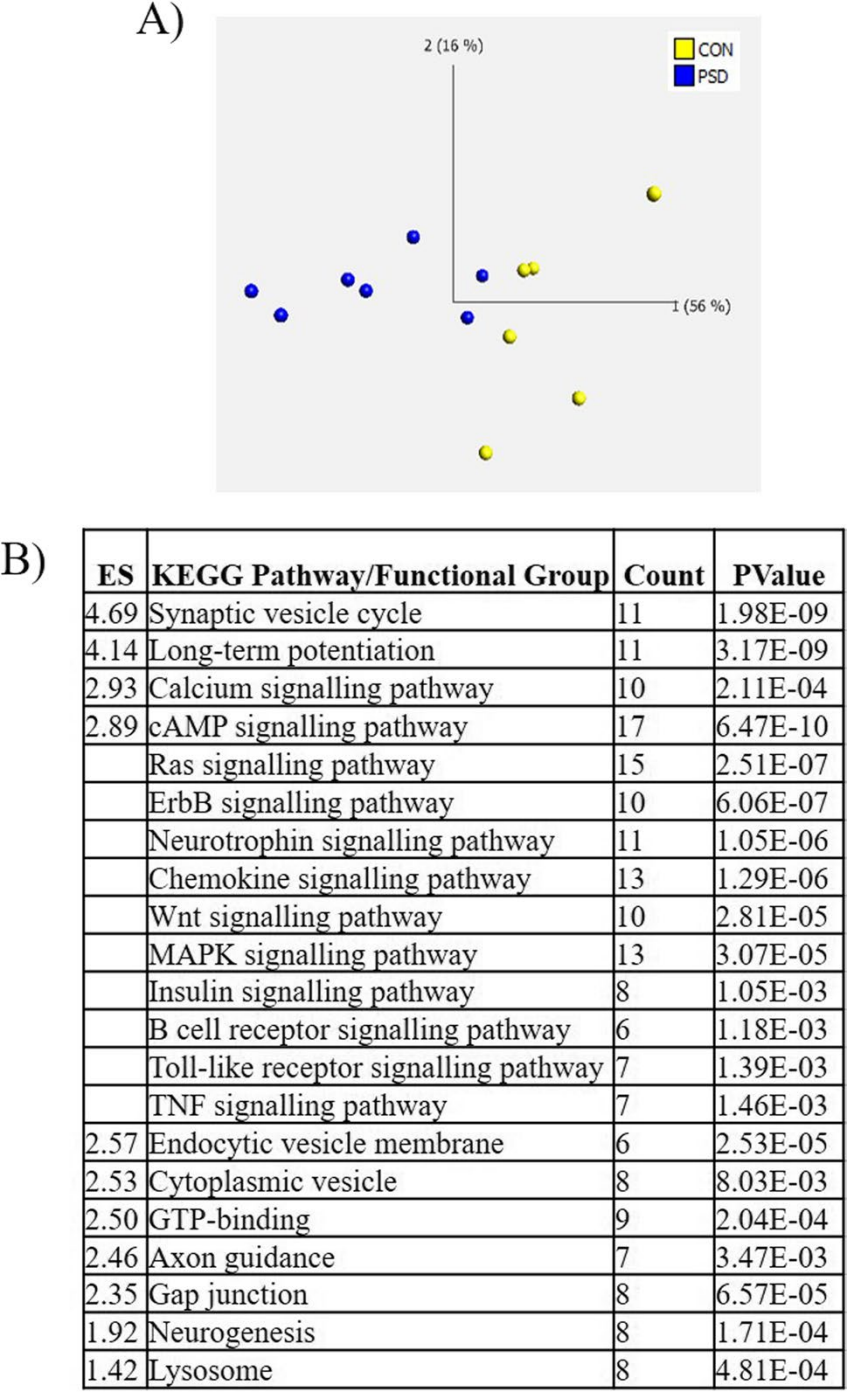
NanoString nCounter gene expression validation of neuronal microarray data. Principal component analysis (PCA) reveals clear separation of poststroke dementia (PSD) and control neuronal cases based on differential gene expression changes analysed via the NanoString nCounter system (A). Functional annotation clustering (FAC) of altered genes analysed via the NanoString nCounter system in PSD vs control neurons (B). (ES; enrichment score)

Validation candidate genes were selected from the initial microarray analysis based on the significant KEGG pathways, FAC and specific, exclusive gene changes associated with PSD neurons versus control neurons. In total, NanoString nCounter analysis showed 97/770 genes on the neuropathology panels to be significantly altered in PSD neurons compared to control neurons (Supplementary Table 7). Based on neuronal gene expression alone, a clear separation of the different patient groups was confirmed using PCA (Fig. 3A). Taking the 97 significantly altered genes (*p* < 0.05), KEGG pathway analysis and FAC showed the same altered pathways that were identified in the microarray study including MAPK signalling (ES; 2.89, 13 genes, p 3.07E-05), Toll-like receptor signalling pathway (ES; 2.89, 7 genes, p 1.39E-03) and Alzheimer’s disease (11 DEGs, p 2.18E-05) in addition to other signalling and synaptic changes (Fig. 3B and Supplementary Table 8).

### Comparison with Neuronal Gene Changes in the BCAS Mouse Model

**Fig. 4:**
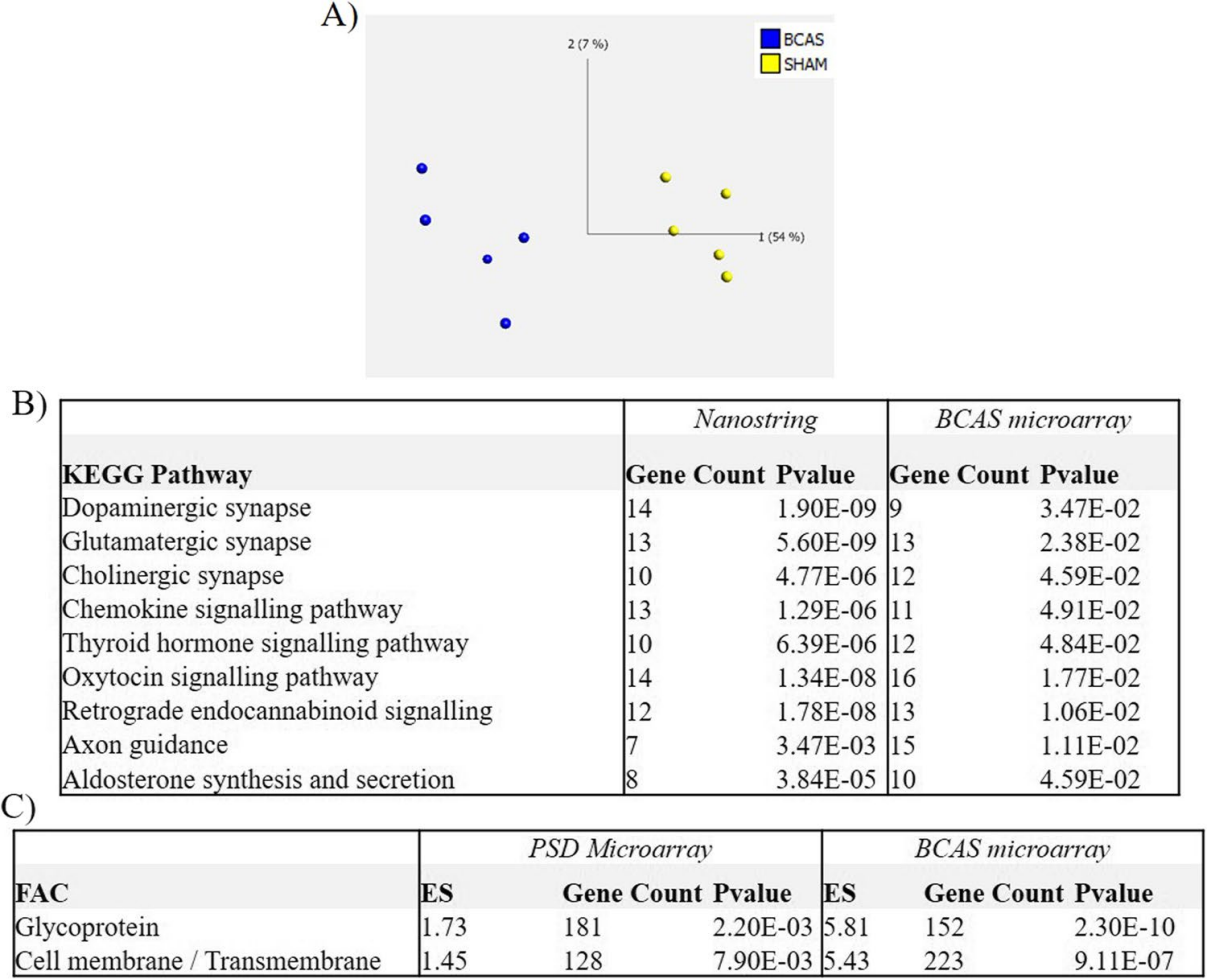
BCAS model neuronal KEGG and FAC analysis. Principal component analysis (PCA) reveals clear separation of BCAS and sham neuronal cases based on differential gene expression changes analysed by microarray analysis (A). KEGG pathway analysis revealed the same synaptic and signalling changes in PSD neurons compared to BCAS neurons (B), while functional annotation clustering (FAC) revealed the same enriched gene clusters between the PSD and BCAS neuronal microarray studies (C) (BCAS, bilateral common carotid artery stenosis; ES, enrichment score)

We also determined whether the changes associated with PSD in human autopsy tissue were found in a mouse model of chronic cerebral hypoperfusion (CCH) that simulates features of vascular cognitive impairment and vascular dementia. Quality control measures were applied to the mouse microarray dataset in TAC, and based on neuronal gene expression alone, a clear separation of the BCAS and sham animal groups was confirmed using PCA (Fig. 4A and Supplementary Table 9). A total of 1185 DEGs (FC ≥ 1.2, ≤ p0.05) were identified between the BCAS and sham group (575 upregulated, 610 downregulated). Following KEGG pathway analysis, several synaptic and signalling pathways were identified in the neuronal BCAS data that were identified in the human PSD neuronal NanoString validation including the downregulation of the dopaminergic synapse (9 DEGs, p 3.47E-02), altered glutamatergic synapse (13 DEGs, p 2.38E-02) and cholinergic synapse (12 DEGs, p 4.59E-02), alongside altered chemokine signalling (11 DEGs, p 4.91E-02), thyroid hormone signalling (12 DEGs, p 4.84E-02), oxytocin signalling (16 DEGs, p 1.77E-02) and retrograde endocannabinoid signalling (13 DEGs, p 1.06E-02) (Fig. 4B). Additional FAC displayed glycoprotein (ES: 5.81, 152 genes, p 2.30E-10) and transmembrane (ES: 5.43, 223 genes, p 9.11E-07) clusters of genes in the neurons isolated from the BCAS model similar to the initial human neuronal microarray findings that showed the same enriched clusters in PSD neurons: glycoprotein (ES; 1.73, 181 genes, p 2.20E-03) and cell membrane (ES: 1.45, 128 genes, p 7.90E-03) (Fig. 4C) (Supplementary Table 10).

### Astrocytic and Endothelium Dysfunction in the White Matter Associated with Poststroke Dementia

Because changes in frontal white matter may also be important in the pathogenesis of dementia poststroke, and may affect neurons, we identified PSD-associated transcriptomic changes in astrocytes and capillary endothelial cells of this area.

**Fig. 5:**
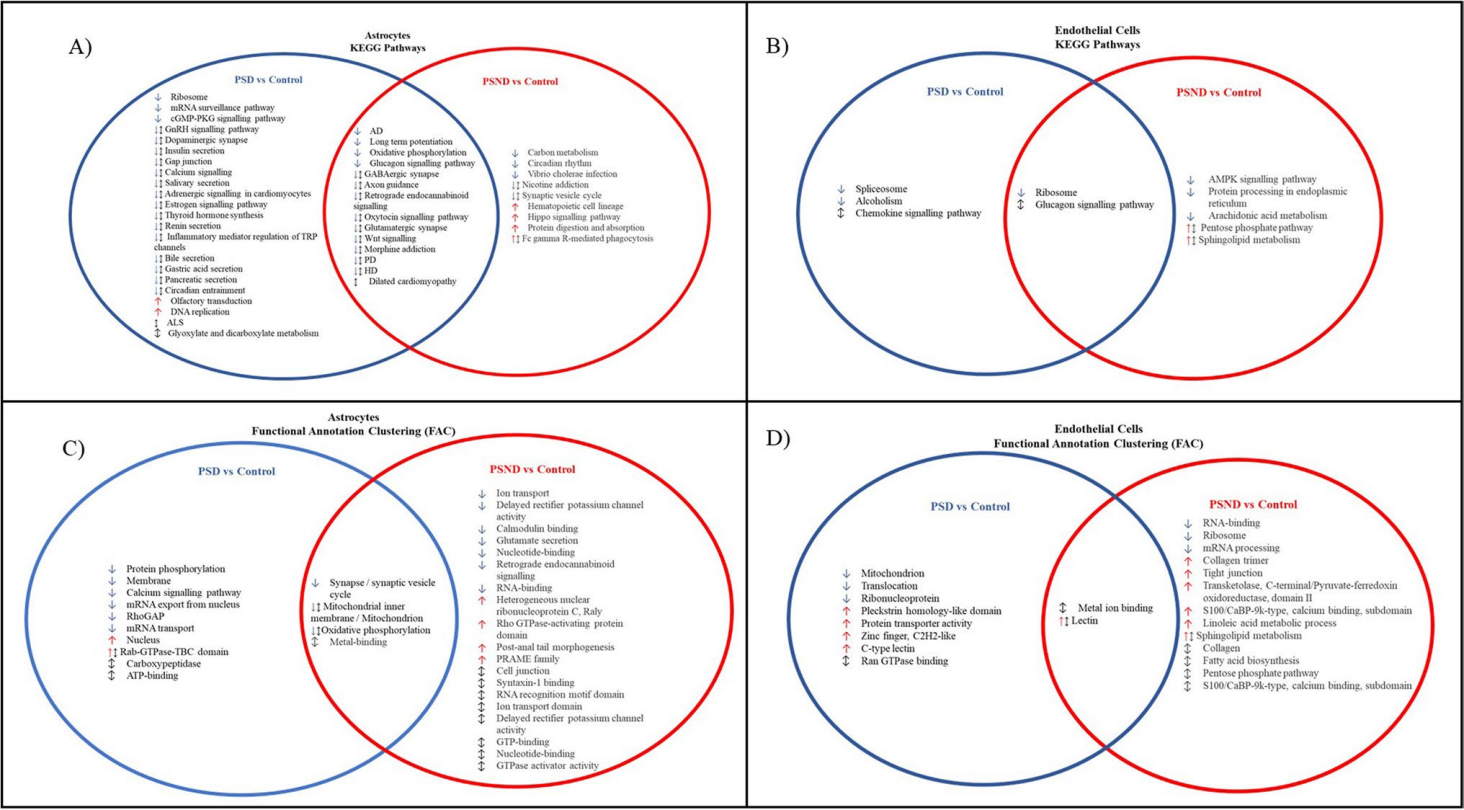
KEGG Pathway analysis and functional annotation clustering associated with having dementia resulting from a stroke (PSD vs. control) compared to changes associated with having a stroke (PSND vs. control) in astrocytes (A&B) and endothelial cells (C&D) (↓ downregulated gene list, ↑ red arrow, upregulated gene list, ↕ up- and downregulated gene lists). (PSD; poststroke dementia, PSND; poststroke non-dementia)

As a result of the stroke, PSND astrocytes showed 767 DEGs compared to control astrocytes. Employing KEGG pathway analysis and FAC, DEGs were associated with changes in metabolism including altered proteolysis, RNA metabolism and phosphorylation alongside changes in Alzheimer’s disease and Parkinson’s disease pathways mostly associated with metabolic gene changes (Fig. 5A and B).

Overall PSD astrocytes showed the greatest number of gene changes compared to control astrocytes (817 DEGs). Astrocytes in the PSD group further showed the greatest number of altered KEGG pathways with most of these pathways downregulated (Fig. 5A). An overall loss of cellular communication was apparent in astrocytes as seen through impaired gap junctions (10 DEGs, p 2.56E-03) and axonal guidance (10 DEGs, p 2.60E-02), alongside both intra- and extracellular signalling changes with altered calcium signalling (18 DEGs p 9.25E-05), glucagon signalling (10 DEGs, p 5.66E-03) and Wnt signalling (10 DEGs, p 4.12E-02). Astrocytic neurotransmitter functions were also altered including changes in dopaminergic synapse (11 DEGs, p 1.04E-02), GABAergic synapse (13 DEGs, p 1.87E-02) and glutamatergic synapse (12 DEGs, p 1.42E-03). The altered KEGG pathways in PSD astrocytes showed downregulation of mitochondria-related genes which was also identified as the most enriched cluster of functionally similar genes in DAVID using FAC (mitochondrial inner membrane, ES: 6.13, 41 genes, p 4.00E-09) alongside changes in mRNA transport (mRNA export from nucleus, ES: 1.50, 10 genes, p 5.90E-03, mRNA transport, ES 1.39, 10 genes, p 5.10E-03) in addition to synapse and membrane clusters (membrane, ES 1.62, 274 genes, p 0.0051; synapse ES:2.44, 25 genes, p 2.70E-04) (Fig. 5B).

Endothelial cells showed the greatest number of DEGs in PSND compared to control cases (1048 DEGs). Through KEGG pathway analysis and FAC, changes in endothelial cell lipid and carbohydrate metabolism were most apparent as a result of a stroke (Fig. 5C and D). Overall, in PSD endothelial cells compared to control endothelial cells, 681 DEGs were identified; through KEGG pathway analysis, an upregulation of chemokine signalling (18 genes, p 5.40E-02) and changes in glucagon signalling (14 genes, p 4.06E-02) (Fig. 5C) were most apparent alongside altered mitochondria, again the most enriched cluster of functionally similar genes in DAVID being the mitochondrion (ES: 3.34, 41 genes, p 5.20E-05) (Fig. 5D).

## Discussion

Progressive dementia after stroke is associated with pathological changes in DLPFC neurons and frontal white matter. As these changes can occur with small strokes not located in situ or in regions of the frontal lobe we assessed, it suggests that such strokes initiate a pathogenic process remote from site of ischemic injury. To elucidate this process further, we investigated transcriptomic changes in DLFPC neurons, and in astrocytes and endothelium of frontal white matter. To identify transcriptomic changes specific to those that develop dementia following a stroke rather than changes due to the stroke itself, we identified changes that were seen in the PSD vs control comparison but were not present in the PSND vs control comparison. In PSD DLPFC neurons, we identified changes in key pathways and genes associated with mitochondria, cellular communication, signalling and metabolism. The key neuronal pathway changes were validated using NanoString nCounter technology, and further experimental validation was completed on neurons isolated from the BCAS mouse model of chronic cerebral hypoperfusion. We also identified in PSD frontal white matter changes in astrocytes signalling and cellular communication, while frontal white matter endothelium showed changes in mitochondria and chemokine signalling pathways. Neuronal atrophy within the DLPFC, rather than neuronal loss, has been shown in PSD [7]. This study, which demonstrates significant dysregulation of numerous neuronal functions including cellular communication, signalling and metabolism, provides further evidence of DLPFC neuronal dysfunction as an important component of PSD pathogenesis, with pathway changes reminiscent of those in neurodegenerative states.

Neuronal endocytosis was dysregulated in the current study with neuronal physiology relying on the interplay between endocytosis and autophagy, as a method of communicating with the environment and maintaining cellular homeostasis [21]. Defects in endocytosis have been identified as a common theme in neurodegenerative diseases including Alzheimer’s disease [22], Parkinson’s disease [23], amyotrophic lateral sclerosis [24] and Huntington’s disease [25] often underlying the earliest events in neurodegeneration [21]. Endocytosis may prevent neurodegeneration by regulating protein turnover via autophagy. Proteins involved in endocytosis, including clathrin-mediated endocytosis (CME) adapters, assembly protein complex 2 (AP-2) and CALM2, control autophagy and regulate the degradation of components of amyloidosis in Alzheimer’s disease [26, 27]. The dysregulation of neuronal endocytosis in PSD as identified in the current study was not identified in those that did not progress to dementia (PSND) which may suggest a dysregulation in endocytosis could contribute to neuronal dysfunction and cognitive decline.

Mitogen-activated protein kinases (MAPKs) are typically activated through extracellular stresses including oxidative stress in addition to other stimuli such as hormones and cytokines and have been extensively studied in association with inflammation [28, 29]. Many of the neuronal genes in the MAPK signalling pathway in the current study were upregulated reinforcing this statement. Seen as a potential mechanism of Alzheimer’s disease pathology, neuronal apoptosis is induced partly by the activation of MAPK signalling pathways [30–32] and human post-mortem studies have identified increased MAPK phosphorylation associated with amyloid beta plaques and neurofibrillary tangles [33]. MAPK signalling is needed for inducing synaptic plasticity [34] alongside brain-derived neurotrophic factor (BDNF), which also plays an important role in neural plasticity and brain recovery following stroke [35]. Interleukin-1 (IL1) is a family of major proinflammatory cytokines, with IL1β previously connected to the neurodegenerative effect of neuroinflammation on cognition and synaptic plasticity in the brain [36]. Chronic elevation of IL1β suppresses BDNF and is a potential therapeutic target in the treatment and recovery from ischaemic stroke [37]. This concept is corroborated in the current study with the upregulation of another IL1 cytokine, *IL1α* and corresponding downregulation of *BDNF* in PSD neurons. It has been reported that IL1β functions in suppressing the effect of BDNF on synaptic plasticity through the induction of p38 MAPK on central nervous system (CNS) neurons [38–40]. The dysregulation of MAPK signalling in PSD neurons and what effect this may have on driving dementia following a stroke requires further investigation.

In the current study, neuronal Toll-like receptor (TLR) signalling was altered in PSD. During brain injury, such as in stroke, the BBB is breached leading to a dysregulation in communication and signalling between the immune system and the CNS [6, 41, 42]. TLRs play a role in the innate immune system by responding to pathogen-derived and tissue damage-related ligands. The interaction between neuronal TLRs and the immune system is well known with alterations in neuronal TLRs having been reported in several neurodegenerative diseases with varying effects [43]. For example, targeting the TLR4/NF*κ*B pathway downregulates the proinflammatory response in both a mouse model of Alzheimer’s disease [44] and traumatic brain injury [45]. Despite not being directly studied in relation to neurodegenerative disease, *TLR3*, known to recognise double-stranded RNA associated with viral infection, was downregulated in the current study [43]. In vivo data collected from early life studies may suggest that neuronal TLR3 stimulation is detrimental in neurodegenerative disease, especially in the context of viral infections including Epstein-Barr virus and hepatitis C [46]. Therefore, identifying whether the dysregulation of neuronal TLR signalling in PSD mediates neuroinflammation and neurodegeneration or forms part of a neuroprotective preconditioning of the brain to respond and handle insult better needs to be established [47].

The current study also identified altered neuronal metabolism in PSD. The TCA cycle functions in the mitochondrial matrix and connects multiple catabolic and anabolic pathways, such as glycolysis and gluconeogenesis that result in the release of stored energy in cells [48]. The decrease in neuronal TCA cycle genes in PSD alongside altered neuronal mitochondrial function may infer a potential pathogenic avenue for neuronal dysfunction. CCH models, which include the BCAS mouse model, have shown energetic deficits reflected by changes in energy metabolites including ATP, adenosine diphosphate (ADP) and adenosine monophosphate (AMP) alongside alteration of ATP-related enzymes [49]. Additionally, these CCH models show mitochondria dysfunction because of decreased cerebral blood flow. Mitochondria also play a role in regulating calcium storage [50], innate and adaptive immunity [51], activating apoptosis [52] and signalling pathways [50]. Altered neuronal mitochondria could serve as a master regulator of the pathology seen in PSD and is worth further study.

To further explore the link between neuronal changes and vascular processes, we also investigated frontal neuronal transcriptome alterations in the BCAS mouse model. The BCAS model is a CCH injury presenting with glial responses and GVU disruption associated with long-term white matter damages accompanied by cognitive decline and is commonly used as an experimental model for vascular dementia [20, 53, 54]. Neuronal changes associated with cell membrane and glycoprotein were remarkably validated in the experimental model, as also key pathway changes including chemokine signalling, axon guidance alongside additional neuronal synaptic changes associated with neuronal dysfunction. In the BCAS mice, similar changes in neurons were identified as seen in PSD neurons, and this strengthens the notion that neuronal degenerative processes maybe linked to vascular processes. As the BCAS models show some of the pathway changes observed in PSD, it will be an important system to investigate aspects of poststroke pathophysiology relevant to the development of dementia.

Previous histological frontal white matter gliovascular alterations involving astrocytes and endothelial cells suggest they are associated with the neuronal changes observed in PSD [6]. We identified an overall dysregulation of astrocyte communication with altered astrocyte calcium signalling in PSD. Astrocyte calcium signalling has a functional role in astrocyte-neuron communication [55, 56], with dysregulated astrocyte calcium signalling occurring as Alzheimer’s type pathology develops [57]. During synaptic activity, the release of neuronal transmitters leads to changes in astrocyte function including calcium activity [58] and these astrocytic calcium signalling changes determine the release of other neuroactive molecules including gliotransmitters: glutamate, GABA and ATP [59]. As a result of a stroke (i.e. PSND vs controls), our study shows a general alteration in astrocytic synaptic effect, yet calcium signalling dysregulation is only apparent in PSD, corroborating the potential importance of modulating astrocytic calcium signalling in PSD that warrants further investigation.

The main endothelial change in PSD was altered chemokine signalling, suggesting immune activation that corroborates the gliovascular histological findings in PSD. The BBB not only protects and maintains homeostasis of the CNS, but also allows tightly controlled communication with the periphery. In multiple sclerosis, brain endothelial cells lose their protective function leading to increased BBB permeability, neuroinflammation and neurodegeneration [60]. Similarly, schizophrenia has been linked to endothelial dysfunction and inflammation [61]. Overall, within frontal white matter, PSD is associated with gliovascular dysfunction, altered astrocyte communication and immune changes in endothelium creating an overall disrupted immune environment. Although it is not possible to pinpoint the sequence of events that lead to PSD from the current study, it may be hypothesised that gliovascular changes in the white matter cause a lack of axonal process support or that frontal white matter neuroinflammation could directly damage axons. Either scenario could result in an impaired ACC and overtime a deterioration in neuronal processing leading to PSD.

**Fig. 6:**
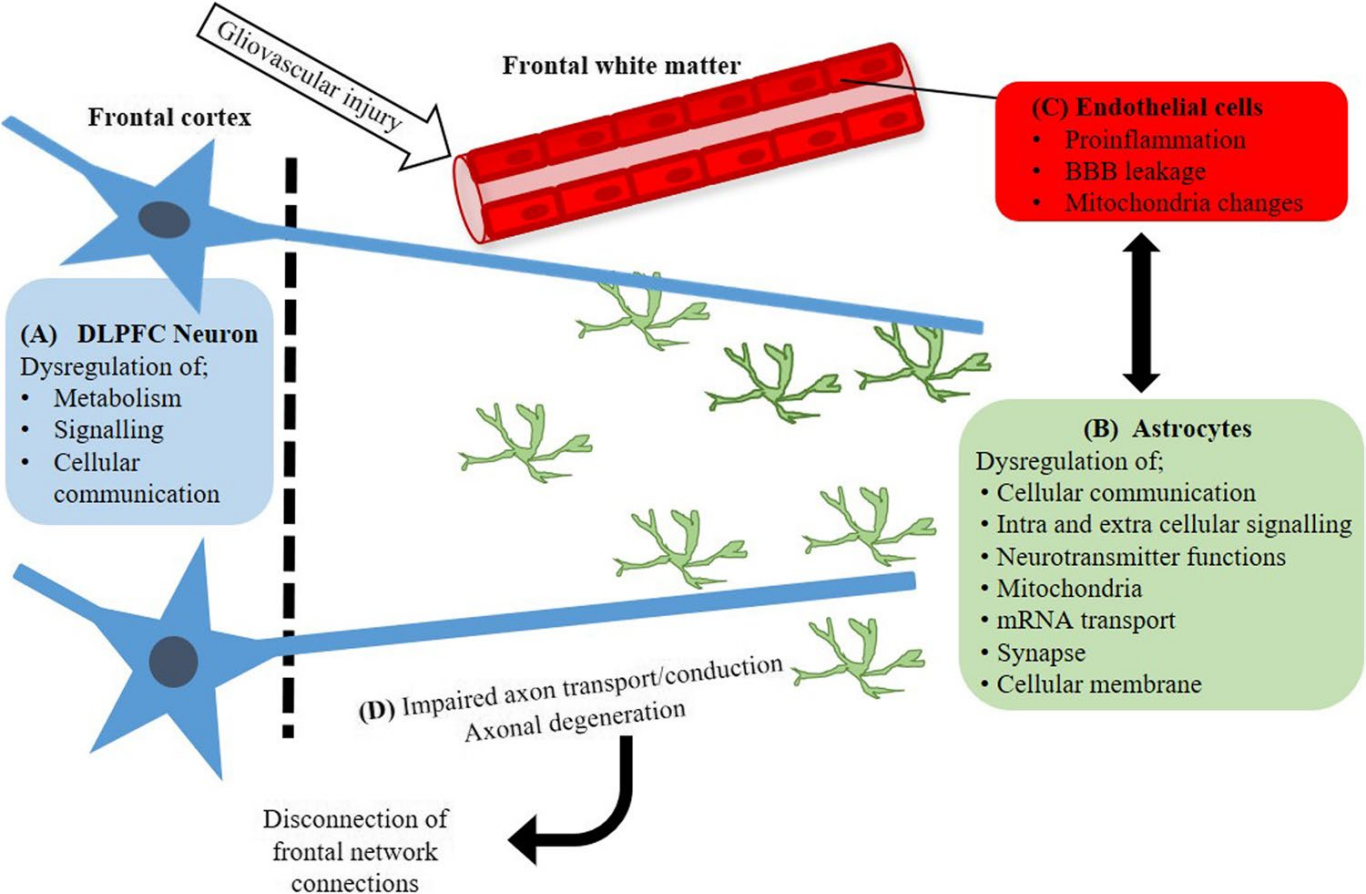
PSD model of pathology in poststroke dementia (PSD)-specific neuronal changes in the dorsal lateral prefrontal cortex (DLPFC) consisted of dysregulation of metabolic processes (TCA cycle, glycoprotein, ATP binding), signalling (MAPK signalling, Toll-like receptor signalling, chemokine signalling, thyroid hormone signalling, oxytocin signalling and retrograde endocannabinoid signalling) and cellular communication (endocytosis, nucleotide binding, cadherin binding, rRNA binding and DNA binding) (A). These PSD neuronal changes within the DLPFC may result from gliovascular changes within the frontal white matter that arise through pathogenic changes resulting from a remote stroke. Astrocytes in PSD showed the greatest number of altered pathways related to cellular communication (gap junction, membrane), signalling (calcium signalling, oestrogen signalling, GnRH Signalling) and neurotransmitter function (dopaminergic synapse, ATP binding) (B). These astrocytic changes were also corroborated by the changes observed in the endothelial cells of blood vessels with altered mitochondria and inflammatory response underpinning an overall loss of frontal white matter gliovascular unit function (C). Ultimately, these changes we propose result in astrocyte failure to support axonal processes that form the cognitive network responsible for executive function (D). Consequently, over time, this would lead to a disconnection of the frontal network resulting in DLFPC neuronal changes apparent in PSD

These transcriptomic findings, and the previous histological studies, propose a possible pathogenic model (Fig. 6) that indicates that in PSD, neuronal changes in the DLPFC consist of a dysregulation of cellular communication, signalling and metabolic processes. These neuronal changes may result from gliovascular changes within the frontal white matter that arise through pathogenic changes resulting from a remote stroke. Thus, in PSD, the GVU in the frontal white matter becomes dysfunctional resulting in altered vascular permeability, neuroinflammation and an overall astrocyte failure in supporting axonal processes that pass through the frontal white matter that form the ACC.

Transcriptomic work using LCM and microarray analysis has limitations. Firstly, transcriptomic analysis is a powerful hypothesis generating tool, but our study has not linked this to proteome changes, while any temporal sequences or functional outcomes may be inferred require further experimental investigation in model systems. Secondly, the current study investigated DLPFC neurons and frontal white matter astrocytes and endothelial cells, yet other cell types including other glial cells such as microglia and oligodendrocytes as well as other cells involved in the GVU including pericytes will undoubtedly play a role in the pathology and must be considered. Thirdly, LCM does not isolate specific cell populations; rather the method allows for an enriched population of cells to be isolated [14]. Therefore, the population of endothelial cells isolated is likely to include pericytes and astrocytic end-feet, yet still gives an assessment of GVU changes.

In conclusion, this study identified key cellular processes that are affected in atrophic DLPFC neurons in PSD. These neuronal pathways share common links to neurodegeneration suggesting a neurodegenerative process in those neurons that underlie the dementia seen in PSD. This may in turn appear related to the changes seen in the frontal white matter. Understanding these changes may allow better prediction of individuals who will develop dementia poststroke, while targeting these neurodegenerative cellular processes could potentially ameliorate this progressive dementia.

## Supplementary Information

Below is the link to the electronic supplementary material.

Supplementary file1 (DOCX 14.3 kb) Supplementary Methods 1

Supplementary file2 (DOCX 28 kb) Supplementary Table 1

Supplementary file3 (DOCX 24 kb) Supplementary Table 2

Supplementary file4 (DOCX 18 kb) Supplementary Table 3_Validation cohort

Supplementary file5 (XLSX 778 kb) Supplementary Table 4_ Full microarray DEG list

Supplementary file6 (XLSX 28 kb) Supplementary Table 5_KEGG pathway analysis

Supplementary file7 (XLSX 38 kb) Supplementary Table 6_ FAC analysis

Supplementary file8 (XLSX 31 kb) Supplementary Table 7_NanoString analysis raw data

Supplementary file9 (XLSX 22 kb) Supplementary Table 8_ Neuron Validation KEGG and FAC analysis

Supplementary file10 (XLSX 98 kb) Supplementary Table 9_BCAS microarray DEG list

Supplementary file11 (XLSX 24 kb) Supplementary Table 10_ BCAS KEGG and FAC analysis

Supplementary file12 (JPG 1002 kb) Supplementary Figure 1: Laser capture microdissection cell isolation enrichment. Endothelial cells of vessels were identified by rapid Coll-V immunostaining. The laser is fired causing the film to melt and adhere to the underlying cell (A). The cap is lifted off with the adhered captured cells (B) leaving behind the remaining tissue (C). For LCM of neurons and astrocyte see references 52 and 58. RT-PCR analysis of cells isolated by immuno-LCM (D). Toluidine blue positive neurons are associated with high levels of NFL transcripts; GFAP positive astrocytes are associated with high levels of GFAP transcripts. Coll-IV positive vessels are associated with high levels of vWF transcripts. Key: Astro, astrocytes; GFAP, glial fibrillary acidic protein; LCM: laser capture microdissection; NFL, neurofilament light; vWF, von willibrand factor.
