# Supplementary material for: Transcriptomic Profiling Reveals Discrete Poststroke Dementia Neuronal and Gliovascular Signatures
Source: Transl Stroke Res. 2022 May 31;14(3):383–96. doi: 10.1007/s12975-022-01038-z (PMC10160172; doi:10.1007/s12975-022-01038-z)
Supplement: Supplementary file 1 — Supplementary file1 (DOCX 14.3 kb) Supplementary Methods 1 [file 12975_2022_1038_MOESM1_ESM.docx]

Transcriptomic profiling reveals discrete post-stroke dementia neuronal and gliovascular signatures

Translational Stroke Research

**Rachel Waller,** Yoshiki Hase, Julie E. Simpson, Paul R. Heath, Matthew Wyles, Rajesh N. Kalaria, Stephen B. Wharton

**Corresponding author affiliation:** Sheffield Institute for Translational Neuroscience, University of Sheffield, Sheffield, S10 2HQ, UK.

**Corresponding author email:** R.Waller@sheffield.ac.uk

**Supplementary Methods 1**

**Animals and surgical procedures**

Male C57BL/6 J mice (9 weeks old, 23−25 g) were purchased from Charles River, UK. The mice were housed confined to a 12-h day and 12-h night cycle (7am– 7pm, day; 7pm–7am, night) and were given access to food and water *ad libitum*. After one week acclimatisation, mice were randomly assigned to either bilateral common carotid artery stenosis (BCAS) or sham surgery. The BCAS surgery was performed as previously described.^15,49^ Briefly, mice were anaesthetised by 1.5 % isoflurane in oxygen and air. A middle neck incision was made, the common carotid arteries (CCAs) were then exposed and isolated from the Vagus nerves. Microcoils, diameter of internal lumen: 0.18 mm (Sawane Spring, Japan), were applied to both CCAs. Sham animals were exposed to the same operative procedures as BCAS mice, except for the application of microcoils. Body temperature was monitored and maintained between 36.5 and 37.5 °C with the aid of a feed-back warming pad and a blanket throughout the operation.

All procedures were pre-approved by the Home Office, London, UK based upon ASPA: The Animals (Scientific Procedures) Act1986, UK and performed in accordance with the guidelines stipulated by the ethical committee of Newcastle University and adhering to ARRIVE guidelines. At four months (16 weeks) after surgery, mice were deeply anaesthetised by intraperitoneal injection of sodium pentobarbital (50 mg/kg) and perfused trans-cardially at 20 ml/min with 0.01 M phosphate-buffered saline, pH 7.4. Brains were subsequently dissected and snap frozen in the liquid nitrogen-cooled isopentane and stored long-term at -80°C. As with human frontal lobe tissues, frontal regions from the BCAS model were free of any local infarcts or other obvious ischemic injury.
