# Supplementary material for: Transcriptomic Profiling Reveals Discrete Poststroke Dementia Neuronal and Gliovascular Signatures
Source: Transl Stroke Res. 2022 May 31;14(3):383–96. doi: 10.1007/s12975-022-01038-z (PMC10160172; doi:10.1007/s12975-022-01038-z)
Supplement: Supplementary file 2 — Supplementary file2 (DOCX 28 kb) Supplementary Table 1 [file 12975_2022_1038_MOESM2_ESM.docx]

Transcriptomic profiling reveals discrete post-stroke dementia neuronal and gliovascular signatures

Translational Stroke Research

**Rachel Waller,** Yoshiki Hase, Julie E. Simpson, Paul R. Heath, Matthew Wyles, Rajesh N. Kalaria, Stephen B. Wharton

**Corresponding author affiliation:** Sheffield Institute for Translational Neuroscience, University of Sheffield, Sheffield, S10 2HQ, UK.

**Corresponding author email:** R.Waller@sheffield.ac.uk

**Supplementary Table 2:** **CogFAST cohort cognitive status**

| **Case ID** | **Age (yr)** | **Gender** | **Last MMSE** | **Last CAMCOG** | **Path Diagnosis** | **VICCCS Diagnosis** | **Dementia status** | **Vascular path Score** | **Infarcts (total #)** | **CERAD** | **Braak Staging** | **CAA** |  |
| --- | --- | --- | --- | --- | --- | --- | --- | --- | --- | --- | --- | --- | --- |
| 1-Con | 72 | M | n/a | n/a | Control | n/a | No dementia | n/a | none | n/a | n/a | n/a |  |
| 2-Con | 78 | F | n/a | n/a | Control | n/a | No dementia | n/a | none | n/a | n/a | n/a |  |
| 3-Con | 72 | F | n/a | n/a | Control | n/a | No dementia | n/a | none | n/a | n/a | n/a |  |
| 4-Con | 74 | F | n/a | n/a | Control | n/a | No dementia | n/a | none | n/a | n/a | n/a |  |
| 5-Con | 74 | F | n/a | n/a | Control | n/a | No dementia | n/a | none | n/a | n/a | n/a |  |
| 6-Con | 94 | F | n/a | n/a | Control | n/a | No dementia | n/a | none | n/a | n/a | n/a |  |
| 7-Con | 78 | F | n/a | n/a | Control | n/a | No dementia | n/a | none | n/a | n/a | n/a |  |
| 8-Con | 89 | F | n/a | n/a | Control | n/a | No dementia | n/a | none | n/a | n/a | n/a |  |
| 9-Con | 73 | M | n/a | n/a | Control | n/a | No dementia | n/a | none | n/a | n/a | n/a |  |
| 10-Con | 96 | F | n/a | n/a | Control | n/a | No dementia | n/a | none | n/a | n/a | n/a |  |
| 1-PSND | 81 | F | 27 | 88 | PSND | n/a | No dementia | 9 | 2 | 2 | 1 | 1 |  |
| 2-PSND | 88 | M | 25 | 87 | PSND | n/a | No dementia | 11 | 2 | 2 | 1 | 2 |  |
| 3-PSND | 83 | F | 26 | 84 | PSND | n/a | No dementia | 14 | 1 | 1 | 2 | 3 |  |
| 4-PSND | 84 | M | 28 | 89 | PSND | n/a | No dementia | 11 | 2 | 2 | 3 | 1 |  |
| 5-PSND | 78 | F | 29 | 99 | PSND | n/a | No dementia | 7 | 2 | 0 | 1 | 0 |  |
| 6-PSND | 82 | M | 26 | 86 | PSND | n/a | No dementia | 13 | 2 | 2 | 4 | 1 |  |
| 7-PSND | 85 | M | 27 | 88 | PSND | n/a | No dementia | 14 | 2 | 0 | 1 | 0 |  |
| 8-PSND | 86 | F | 25 | 88 | PSND | n/a | No dementia | 14 | 2 | 1 | 4 | 0 |  |
| 9-PSND* | 92 | F | 4 | n/a | PSND | VaD | Dementia- severe | 15 | 2 | 0 | 2 | 0 |  |
| 10-PSND | 99 | M | 25 | 85 | PSND | n/a | No dementia | 12 | 2 | 0 | 2 | 0 |  |
| 1-PSD | 96 | M | 20 | 65 | PSD | VaD | Dementia | 14 | 8 | 1 | 2 | 0 |  |
| 2-PSD | 88 | F | 16 | 71 | PSD | VaD | Dementia | 14 | 11 | 0 | 1 | 1 |  |
| 3-PSD | 82 | M | 16 | 62 | PSD | VaD | Dementia | 13 | 9 | 0 | 0 | 0 |  |
| 4-PSD | 93 | M | 12 | 24 | PSD | VaD | Dementia - severe | 16 | 6 | 2 | 3 | 1 |  |
| 5-PSD | 87 | F | 6 | 47 | PSD | VaD | Dementia - severe | 14 | 6 | 1 | 3 | 1 |  |
| 6-PSD | 75 | M | 0 | 0 | PSD | VaD | Dementia - severe | 15 | 11 | 0 | 0 | 0 |  |
| 7-PSD | 89 | F | 16 | 65 | PSD | VaD | Dementia | 17 | 1 | 2 | 3 | 1 |  |
| 8-PSD | 96 | M | 13 | 48 | PSD | VaD | Dementia- severe | 16 | 7 | 0 | 3 | 2 |  |
| 9-PSD | 97 | F | 17 | 63 | PSD | VaD | Dementia | 15 | 2 | 1 | 1 | 1 |  |
| 10-PSD | 91 | M | 12 | 55 | PSD | VaD | Dementia- severe | 12 | 5 | 0 | 3 | 0 |  |

*Deficits mainly in expression rather than cognition

Vascular Pathology Score [2]: 1-20;

Infarcts size <5mm: # total number.

CERAD (Abeta load score) [5,6]: 0 = no neuritic plaques, 1 = sparse neuritic plaques, 2 = moderate neuritic plaques, 3 = frequent neuritic plaques

Braak [1]: 0-6 = Braak stages for Alzheimer's disease-type neurofibrillary pathology

CAA: 0 = no CAA, 1 = mild, focal CAA, 2 = moderate CAA, 3 = severe CAA

CAMCOG [3,7]: Scores less than 80 indicative of dementia

MMSE [4]: Scores >24+ normal cognition, no dementia; 19-23 mild dementia; 10-18; moderate dementia; <9 severe dementia

**Key:** CAA: Cerebral amyloid angiopathy; CAMCOG: Cambridge Cognition Examination; CERAD: Consortium to Establish a Registry for Alzheimer's Disease; Con: Control; F: Female; M: Male; MMSE: Mini-Mental State Exam; n/a: data not available/not applicable; PSD: Post-stroke dementia; PSND: Post-stroke non dementia; VaD: Vascular dementia; VICCCS: Vascular Impairment of Cognition Classification Consensus Study; yr: year.

**References**

1. Braak, H., Braak, E. (1991) Neuropathological stageing of Alzheimer-related changes. Acta Neuropathol 82, 239–259. Doi.org/10.1007/BF00308809
2. Deramecourt V, Slade JY, Oakley AE, et al. (2012) Staging and natural history of cerebrovascular pathology in dementia. Neurology. 78(14):1043-1050. Doi:10.1212/WNL.0b013e31824e8e7f
3. de Koning, I., van Kooten, F., Dippel, D.W.J., van Harskamp, F., Grobbee, D.E., Kluft, C. & Koudstaal, P.J. (1998) The CAMCOG: A useful screening instrument for dementia in stroke patients. Stroke, 29, 2080-2086. Doi.org/10.1161/01.STR.29.10.2080
4. Folstein MF, Folstein SE, McHugh PR. (1975) “Mini-mental state”. A practical method for grading the cognitive state of patients for the clinician. Journal of Psychiatric Research 12(3):189–98. Doi: 10.1016/0022-3956(75)90026-6
5. Mirra S, Heyman A, McKeel D, Sumi SM, Crain BJ, Brownlee LM, et al. (1991) The Consortium to Establish a Registry for Alzheimer’s Disease (CERAD). Part II. Standardization of the neuropathologic assessment of Alzheimer’s disease. Neurology 41:479–86. Doi: 10.1212/wnl.41.4.479
6. Morris JC, Heyman A, Mohs RC, Hughes JP, van Belle G, Fillenbaum G, et al. (1989) The Consortium to Establish a Registry for Alzheimer’s Disease (CERAD). Part 1. Clinical and Neuropsychological Assessment of Alzheimer’s Disease. Neurology 39:1159–65. Doi: 10.1212/wnl.39.9.1159
7. Roth, M., Tym, E., Mountjoy, C., Huppert, F.A., Hendrie, H., Verma, S. et al. (1986) CAMDEX: A standardized instrument for the diagnosis of mental disorder in the elderly with special reference to the early detection of dementia. British Journal of Psychiatry, 149, 698-709. Doi: 10.1192/bjp.149.6.698
