# Supplementary material for: Transcriptomic Profiling Reveals Discrete Poststroke Dementia Neuronal and Gliovascular Signatures
Source: Transl Stroke Res. 2022 May 31;14(3):383–96. doi: 10.1007/s12975-022-01038-z (PMC10160172; doi:10.1007/s12975-022-01038-z)
Supplement: Supplementary file 3 — Supplementary file3 (DOCX 24 kb) Supplementary Table 2 [file 12975_2022_1038_MOESM3_ESM.docx]

Transcriptomic profiling reveals discrete post-stroke dementia neuronal and gliovascular signatures

Translational Stroke Research

**Rachel Waller,** Yoshiki Hase, Julie E. Simpson, Paul R. Heath, Matthew Wyles, Rajesh N. Kalaria, Stephen B. Wharton

**Corresponding author affiliation:** Sheffield Institute for Translational Neuroscience, University of Sheffield, Sheffield, S10 2HQ, UK.

**Corresponding author email:** R.Waller@sheffield.ac.uk

**Supplementary Table 1:** **CogFAST cohort comorbidities and medication history**

|  |  |  |  |  |  |  |
| --- | --- | --- | --- | --- | --- | --- |
| **Case** | **Age (yr)** | **Sex** | **Hypertension**  **(Y/N)** | **Hypertension treatment** | **Smoking history** | **Comorbidities** |
| 1-Con | 72 | M | Y | Atenolol, Bendroflumethiazide | n/a | Arterial fibrillation |
| 2-Con | 78 | F | Y | Frusemide | Y | COPD, IHD |
| 3-Con | 72 | F | Y | Atenolol, Bendroflumethiazide | N | Rheumatoid arthritis |
| 4-Con | 74 | F | N | N | Y | Ulcerative colitis, duodenal ulcer, arthritis, IHD |
| 5-Con | 74 | F | Y | Amlodipine, Frusemide | Y | aortic aneurysm , IBS |
| 6-Con | 94 | F | n/a | n/a | n/a | n/a |
| 7-Con | 78 | F | Y | n/a | N | Pelvic mass, Ovarian tumour |
| 8-Con | 89 | F | N | n/a | n/a | Asthma, AF, CCF |
| 9-Con | 73 | M | N | n/a | n/a | Cerebral palsy |
| 10-Con | 96 | F | Y | Ramipril, Atenolol, Lercanidipine | n/a | Cancer tonsil and throat. AF. Hypothyroidism |
| 1-PSND | 81 | F | n/a | n/a | Y | Angina, MI |
| 2-PSND | 88 | M | Y | Bendroflumethiazide | n/a | n/a |
| 3-PSND | 83 | F | Y | Bendroflumethiazide | N | n/a |
| 4-PSND | 84 | M | Y | Calcium channel blocker | n/a | n/a |
| 5-PSND | 78 | F | n/a | n/a | n/a | n/a |
| 6-PSND | 82 | M | Y | Bendroflumethiazide, Amlodipine | Y | n/a |
| 7-PSND | 85 | M | Y | Amlodipine, | Y | n/a |
| 8-PSND | 86 | F | n/a | n/a | n/a | n/a |
| 9-PSND | 92 | F | Y | Ace inhibitor | Y | n/a |
| 10-PSND | 99 | M | Y | Atenolol, Lisinopril | n/a | IHD, MI, TIA, Type 2 diabetes, chronic lymphocytic leukaemia. |
| 1-PSD | 96 | M | n/a | n/a | n/a | n/a |
| 2-PSD | 88 | F | Y | Atenolol, Frusemide | N | n/a |
| 3-PSD | 82 | M | n/a | n/a | n/a | n/a |
| 4-PSD | 93 | M | n/a | n/a | n/a | n/a |
| 5-PSD | 87 | F | n/a | n/a | n/a | n/a |
| 6-PSD | 75 | M | N | n/a | N | n/a |
| 7-PSD | 89 | F | n/a | n/a | n/a | Hypothyroid |
| 8-PSD | 96 | M | Y | Calcium channel blocker & Ace inhibitor | n/a | IHD |
| 9-PSD | 97 | F | Y | Beta blocker, Ace inhibitor and diuretic | n/a | Heart failure, AF |
| 10-PSD | 91 | M | n/a | n/a | n/a | AF, IHD, CKD |
|  |  |  |  |  |  |  |

**Key:** AF: Arterial fibrillation; CCF: congestive heart failure; CKD: Chronic kidney disease; COPD: Chronic obstructive pulmonary disease; F: Female; h: hour; IBS: Irritable bowel syndrome; IHD: Ischaemic heart disease; M: Male; MI: Myocardial infarction; n/a: data not available; TIA: Transient ischaemic attack; Y/N: yes/no; yr: year.
