# Supplementary material for: Transcriptomic Profiling Reveals Discrete Poststroke Dementia Neuronal and Gliovascular Signatures
Source: Transl Stroke Res. 2022 May 31;14(3):383–96. doi: 10.1007/s12975-022-01038-z (PMC10160172; doi:10.1007/s12975-022-01038-z)
Supplement: Supplementary file 4 — Supplementary file4 (DOCX 18 kb) Supplementary Table 3_Validation cohort [file 12975_2022_1038_MOESM4_ESM.docx]

Transcriptomic profiling reveals discrete post-stroke dementia neuronal and gliovascular signatures

Translational Stroke Research

**Rachel Waller,** Yoshiki Hase, Julie E. Simpson, Paul R. Heath, Matthew Wyles, Rajesh N. Kalaria, Stephen B. Wharton

**Corresponding author affiliation:** Sheffield Institute for Translational Neuroscience, University of Sheffield, Sheffield, S10 2HQ, UK.

**Corresponding author email:** R.Waller@sheffield.ac.uk

**Supplementary Table 1 CogFAST validation cohort: case number, age, gender, post-mortem delay, brain pH and cause of death**

|  |  |  |  |  |  |
| --- | --- | --- | --- | --- | --- |
|  |  |  |  | **DLPFC sample** | |
| **Case** | **Age (yr)** | **Sex** | **PMI (h)** | **Pre-LCM RIN** | **Neu RIN** |
| 1-Con | 72 | M | 17 | 2.4 | 3.1 |
| 2-Con | 78 | F | 23 | 2.2 | 2.7 |
| 5-Con | 74 | F | 53 | 2.4 | 3.1 |
| 6-Con | 94 | F | 15 | 1.6 | 2.6 |
| 8-Con | 89 | F | 98 | 2.9 | 2.5 |
| 9-Con | 73 | M | 25 | 7.1 | 2.7 |
| 10-Con | 96 | F | 29 | 2.4 | n/a |
| 2-PSD | 88 | F | 71 | 2.4 | 2.5 |
| 3-PSD | 82 | M | 19 | 2.6 | 2.5 |
| 5-PSD | 87 | F | 23 | 2.4 | 2.6 |
| 6-PSD | 75 | M | 24 | 2.4 | n/a |
| 7-PSD | 89 | F | 81 | 2.2 | n/a |
| 9-PSD | 97 | F | 88 | 1.9 | 2.8 |
| 10-PSD | 91 | M | 12 | n/a | n/a |
|  |  |  |  |  |  |

**Key:** Con: control; DLPFC: dorsal lateral prefrontal cortex; F: female; h: hour; M: male; n/a: data not available; Neu: neurons; PMI: post-mortem interval; PSD: post-stroke dementia; RIN: RNA integrity number; yr: year.
