# Supplementary figures and images for: Transcriptomic Profiling Reveals Discrete Poststroke Dementia Neuronal and Gliovascular Signatures
Source: Transl Stroke Res. 2022 May 31;14(3):383–96. doi: 10.1007/s12975-022-01038-z (PMC10160172; doi:10.1007/s12975-022-01038-z)

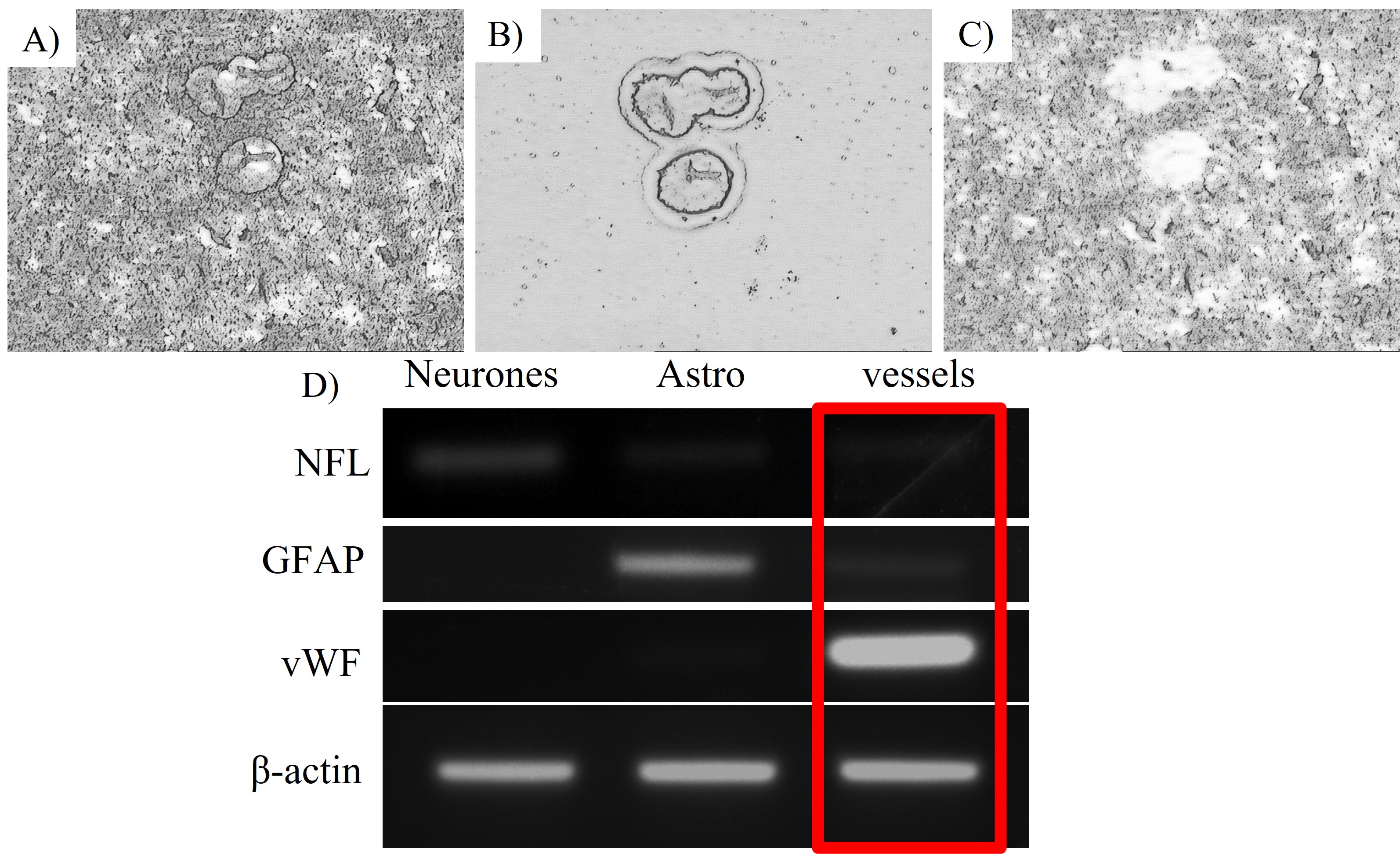

Supplement: Supplementary file 12 — Supplementary file12 (JPG 1002 kb) Supplementary Figure 1: Laser capture microdissection cell isolation enrichment. Endothelial cells of vessels were identified by rapid Coll-V immunostaining. The laser is fired causing the film to melt and adhere to the underlying cell (A). The cap is lifted off with the adhered captured cells (B) leaving behind the remaining tissue (C). For LCM of neurons and astrocyte see references 52 and 58. RT-PCR analysis of cells isolated by immuno-LCM (D). Toluidine blue positive neurons are associated with high levels of NFL transcripts; GFAP positive astrocytes are associated with high levels of GFAP transcripts. Coll-IV positive vessels are associated with high levels of vWF transcripts. Key: Astro, astrocytes; GFAP, glial fibrillary acidic protein; LCM: laser capture microdissection; NFL, neurofilament light; vWF, von willibrand factor. [file 12975_2022_1038_MOESM12_ESM.jpg]
